# Spatial Learning in a Virtual Environment: The Role of Self-Efficacy Feedback and Individual Visuospatial Factors

**DOI:** 10.3390/brainsci11091185

**Published:** 2021-09-09

**Authors:** Laura Miola, Veronica Muffato, Chiara Meneghetti, Francesca Pazzaglia

**Affiliations:** 1 Department of General Psychology, University of Padova, 35131 Padova, Italy; veronica.muffato@unipd.it (V.M.); chiara.meneghetti@unipd.it (C.M.); francesca.pazzaglia@unipd.it (F.P.); 2 Interuniversity Research Centre in Environmental Psychology (CIRPA), 00185 Rome, Italy

**Keywords:** self-efficacy, feedback, spatial learning, individual differences

## Abstract

We examined the roles self-efficacy plays in environmental learning in terms of self-efficacy feedback and task-specific (navigation-based) self-efficacy. We manipulated self-efficacy using positive and neutral feedback to investigate the relationship between receiving positive feedback and environmental learning performance and subsequent recall. A total of 231 participants were administered visuospatial tasks, where 117 received positive feedback, and 114 received neutral feedback. Then, we tested environmental learning using route retracing, pointing, and map-completion tasks. Before each environmental task, participants evaluated their task-specific self-efficacy. A series of spatial self-reported preferences were gathered as well. Mediation models showed that receiving positive feedback after a visuospatial task influences environmental recall performance through the mediation of task-specific self-efficacy. Moreover, after accounting for experimental manipulation and gender, we found that task-specific self-efficacy, sense of direction, and visuospatial abilities influence spatial-recall task performance, even with some differences as a function of the specific recall tasks considered. Overall, our findings suggest that among individual characteristics, task-specific self-efficacy can sustain environmental learning. Furthermore, giving positive feedback can improve spatial self-efficacy before conducting spatial-recall tasks.

## 1. Introduction

Navigating and reaching destinations within an environment are important abilities in people’s daily lives. During navigation, individuals learn various spatial information—for instance, routes, landmarks, directions, and turns [1]—that contribute to creating a mental representation of the environment [2]. It has been highlighted that virtual reality has been increasingly used in experimental psychology over the last 20 years. Indeed, assessing spatial knowledge in virtual environment or virtual reality has the advantage of allowing the control of stimulus presentation, response options, sensory inputs, and scenarios. [3]. Moreover, the validity of the virtual environment increases when it reproduces three-dimensional entities in a fully immersive condition that resembles real-life experience; even the use of a desktop presentation (as it will be used in the current study) is a good approximation for the formation of environmental knowledge [4]. Furthermore, it has been found that the abilities used to learn a real and virtual environment are partially overlapping [5].

Environmental learning is a complex ability with a large variability. Therefore, interest in studying individual spatial factors that explain environmental learning has grown. Individual spatial factors consist of visuospatial cognitive abilities and self-reported spatial abilities that have been highlighted in sustaining the accuracy of environmental learning and spatial mental representations of the environment [5,6]. Among visuospatial cognitive abilities is visuospatial working memory (VSWM) [7], mental rotation abilities, spatial visualization, and spatial perception [8]. In addition to visuospatial cognitive abilities, self-reported evaluations of individuals’ spatial competences include, for instance, spatial sense of direction and spatial anxiety [9]. To clarify the roles of these individual factors, we conducted a study and quantified the effect of individual spatial factors. We found that subjective evaluations and, in particular, visuospatial cognitive abilities relate to accuracy in various spatial-recall measures (route retracing, shortcut finding, and landmark locating) after environmental navigation learning [10].

Regarding self-reported spatial abilities, only recently have motivational factors such as self-efficacy beliefs been studied in the spatial cognition field. Self-efficacy has been thoroughly studied in other areas (e.g., academic motivation, sports performance, and health behaviors). Self-efficacy in general has been conceptualized by Hertzog and Dixon (1994) from the metamemory framework in a hierarchical structure. In fact, self-efficacy can be conceived from a more global and dispositional level (beliefs and individual perception of his or her usual general spatial/memory ability across many situations in various memory domains) to a more task-specific and situational level [11]. Moreover, it has been shown that the perceived ability to perform a specific cognitive task (i.e., task-specific self-efficacy) relates more strongly to a better cognitive performance in memory tasks rather than the perceived general cognitive ability or global self-efficacy [12]). For the abovementioned reasons, it is important to consider both global and task-specific beliefs when assessing self-efficacy. In the spatial cognition domain, Burles et al. (2014) assessed motivational aspects such as general self-esteem (e.g., “I feel that I have a number of good qualities”), general self-efficacy (e.g., “I will be able to successfully overcome many challenges”), and locus of control (e.g., “I believe that my success depends on ability rather than luck”), and they found a positive relationship between self-esteem and spatial performance in a cognitive map-formation task [13]. A few others have also considered the relationship between spatial performance and self-efficacy, but they used a domain-specific, self-efficacy measure regarding spatial abilities and navigation (spatial self-efficacy; [14,15]). Spatial self-efficacy can be defined as the beliefs referring to one’s ability to accomplish spatial tasks. Pazzaglia et al. (2017) showed that spatial self-efficacy, together with the pleasure in exploring, predicts performance in shortcut finding. Participants with higher levels of self-efficacy found shorter paths to reach a destination, especially in complex environments (i.e., virtual environments without landmarks). So far, spatial self-efficacy has been measured using a questionnaire that acquires a global measure of spatial self-efficacy, resulting from self-efficacy estimation referred to several everyday spatial situations (e.g., Wayfinding Self-Efficacy Questionnaire) [14,15]; however, to our knowledge, no studies have investigated task-specific self-efficacy in spatial tasks.

In line with self-efficacy theory, the abovementioned evidence indicates that spatial performance is related to self-efficacy beliefs. Self-efficacy is often considered an antecedent of resource commitment and performance [14,16]. However, self-efficacy can also be influenced by past performance and mastery experience. In other words, when individuals perform better in a task, they become more efficacious. This can in turn influence future beliefs of one’s own efficacy [16]. In addition to mastery experience, another means that could influence self-efficacy is verbal persuasion through feedback. Verbal persuasion, in fact, is considered one of the sources of self-efficacy and is often transmitted through evaluation given to the person who acts [16]. One kind of feedback is normative feedback consisting of giving an individual information on their performance compared to others’. When the normative feedback is positive, the individual indicates above-average performance. Evidence suggests that such positive normative feedback, even if fictitious and independent of a performance’s effectiveness, can sustain subsequent performance [17]. Studies that implemented normative feedback administered various kinds of cognitive tasks (e.g., arithmetic tasks, name recall). They demonstrated that participants who received positive normative feedback reported higher self-efficacy and performed better compared to those who did not receive feedback [18,19]. These results seem to suggest that fictitious positive normative feedback is an effective intervention to promote self-efficacy [18]. However, to our knowledge, no studies have manipulated self-efficacy in the spatial cognition domain.

Our main aim was to investigate whether self-efficacy manipulated through positive feedback (as opposed to neutral feedback) is related to performance in environmental learning and subsequent recall. More specifically, we investigated whether receiving positive feedback on visuospatial performance affects subsequent performance in spatial-recall tasks after environmental learning. The experimental manipulation consisted of giving the experimental group positive feedback after completing three visuospatial tasks and giving neutral feedback to the control group. Then, the same participants were asked to learn a virtual environment based on navigation and to perform spatial-recall tasks that referred to the environment just learned: route retracing to assess navigation ability; and pointing and map location to assess how well participants mentally represented spatial information.

One recent study on arithmetic tasks [18] suggested that the effect of positive feedback on spatial learning is mediated by increasing self-efficacy. Our second aim was to investigate whether positive feedback could increase spatial self-efficacy in spatial-recall tasks. This, in turn, can influence spatial learning performance. We used a mediation analysis to test this relationship. We expected that levels of task-specific self-efficacy would be higher in the group with positive feedback and that task-specific self-efficacy could mediate the effect of the feedback on spatial-recall performance [18].

Our third aim was to investigate whether self-efficacy (both global and task-specific) predicted participants’ performance in recall tasks independently of the feedback condition. We controlled for individual visuospatial individual factors as well. Indeed, after inserting a feedback condition (our manipulation), we added gender and individual spatial factors (visuospatial abilities and self-reported spatial preferences), as have been found related to spatial-recall performance after learning in a virtual environment [10,20]. As the last predictors, we added global and task-specific self-efficacy. We expected task-specific self-efficacy to predict environmental learning [12].

We implemented tasks measuring visuospatial cognitive abilities for the purpose of the manipulation. Together with self-reported measures, such tasks can play a relevant role in predicting spatial-recall task performance after navigation [5,10]. We selected visuospatial tasks so that it was difficult for a given participant to fully understand their own performance and comparison with others (e.g., recording the time taken to complete the tasks). Global spatial self-efficacy was measured using a questionnaire that referred to general environmental situations. Task-specific self-efficacy was measured using a single question just before performing spatial-recall tasks in the virtual environment. These two aspects of self-efficacy can be differently related to cognitive performance.

## 2. Materials and Methods

### 2.1. Participants

A sample of 231 young adults (117 women) aged 18 to 40 were involved in the study (age mean = 23.85; SD = 4.06). All participants volunteered to take part in the experiment and were native Italian speakers. Participants were randomly assigned to two feedback conditions: 117 received positive feedback, and 114 received neutral feedback. The study was conducted in accordance with the recommendations of the local university’s research ethics committee (approval No. 3914) and with the fundamental principles established in the Declaration of Helsinki (2013). All participants were informed about the aims of the study and gave written informed consent before and after the experiment. In the informed consent prior to the research, each participant was told that the purpose of the research was to study the relationship between sense of direction, spatial self-efficacy, spatial anxiety, and learning a virtual environment. At the end of the experiment, the experimenter gave a very detailed debriefing to the participant. In this phase, the aims of the study were resumed and deepened, and both condition groups were told that the fictitious positive feedback given to the experimental group was expected to increase (compared to the control group with neutral feedback) its sense of self-efficacy and spatial performance, as well as determine whether increased self-efficacy corresponded to better performance in subsequent trials. Furthermore, any additional information the participants would require was given, including their actual performance in the initial trials.

### 2.2. Materials

#### 2.2.1. Visuospatial Ability Tasks

The following measures were used to perform basic visuospatial tasks, after which the participants received feedback.

- Route Task [21]

This task aims to evaluate the participants’ ability to switch from a map perspective (allocentric view) to an egocentric perspective. A schematic map with a path outlined inside was presented to participants. Each participant’s task was to imagine tracing the path from a first-person perspective and to describe aloud, as quickly as possible, which directions to take to reach the end point (i.e., go straight, turn right, turn left). Each correct answer was awarded 1 point, for a maximum of 17. During the test, times were recorded, but no time limit was given.

- Survey Task [22]

This task aimed to evaluate the participants’ ability to use allocentric coordinates. Two points (start and end points) joined by a series of segments were presented to participants. Each participant’s task was to judge the distance between the two points, imagining the segment as straightened out and mentally adding the segments’ length for a total of seven trials. In answering each question, participants chose from four alternatives (i.e., straight lines of different lengths). Each correct answer was awarded 1 point, for a maximum score of 7.

- Map Memory [23]

This task aimed to investigate visual memory—that is, the ability to remember configurations of figural stimuli. A total of 12 maps were shown to participants simultaneously. Each participant’s task consisted of carefully watching and memorizing the maps for 3 min. Immediately afterward, a page with another 12 maps was shown to the participant. The latter was required to recognize those they had just studied. The task was composed of two trials. Each correct identification (presence or absence) was scored as 1 point, for a maximum score of 24.

#### 2.2.2. Spatial Self-Assessment Measures

- Wayfinding Self-Efficacy Questionnaire [14]

The items on the self-efficacy questionnaire correspond to items of the Spatial Anxiety Scale) (SAS; [9]). In this case, the questionnaire had different instructions, such as to indicate how well the participants felt able to perform the tasks described. We used this questionnaire to assess global spatial self-efficacy, such as how confident individuals feel about their ability to perform environmental spatial tasks (e.g., finding the right path in an unfamiliar environment). It consisted of eight items scored on a 6-point Likert scale (1 = *not at all* to 6 = *very much*). The maximum score was 48. The internal reliability was good (α = 0.81).

- Task-Specific Spatial Self-Efficacy

Before each environmental task, participants were asked to indicate how well they felt able to accomplish the spatial-recall tasks (i.e., “Now that the task has been explained to you, how well do you feel you can do the task you are about to tackle?”). Participants assigned themselves a score on a scale of 0 to 100 (0 = *not at all* to 100 = *very much*). The scale was based on the guide for constructing self-efficacy scales [24].

- Sense of Direction and Spatial Representation Questionnaire (SDSR; [25])

We used this questionnaire to assess sense of direction and spatial preferences. It is composed of 10 items covering a self-reported sense of direction; a preference for a map-, route-, or landmark-based modality; and knowledge and usage of cardinal points (e.g., “Do you think you have a good sense of direction?”). Answers were given using a 5-point Likert scale (1 = *not at all* to 5 = *very much*). The maximum score was 70. The internal reliability was good (α = 0.82).

- Spatial Anxiety Scale (SAS; [25])

We used this questionnaire to assess the degree of space-related anxiety experienced in an environment. It consisted of eight items scored on a 6-point Likert scale (1 = *not at all* to 6 = *very much*; e.g., “going to an appointment in an unfamiliar part of the city”). Participants’ final score is the sum of their ratings for each item, with higher scores corresponding to greater spatial anxiety. The maximum score was 48. The internal reliability was good (α = 0.81).

#### 2.2.3. Virtual Environment and Recall Measures

- Encoding Phase

**Figure 1:**
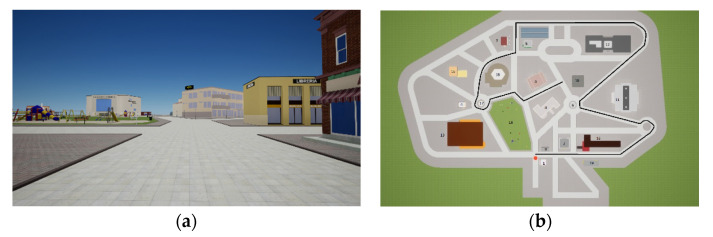
Example route-retracing screenshot (**a**) and sketch map (not shown to participants) with landmarks and the route learned (**b**).

The virtual environment (modeled with Rhino, Unreal Engine Version 4.21) consisted of a virtual city with 19 landmarks (e.g., buildings, a park, a fountain, a sculpture) and streets. Participants learned an approximately 1-km route within the environment that encountered all its landmarks and contains 16 intersections of streets (see Figure 1a,b). Specifically, a video from a first-person perspective was created and shown to the participants twice, with instructions to watch carefully and learn the route, the environment, and all landmarks. The video lasted around 4 min, it was created at an eye height of 160 cm, and the camera was set with a horizontal field of view of 90°. The walking speed was 4 m/s.

- Testing Phase

Route direction task. This task consisted of showing each participant a screenshot representing an intersection of the streets on the previously seen path (see Figure 1). For each screenshot, the participant was required to indicate the direction needed to proceed to retrace the route shown in the video by inserting an arrow inside the screenshot. The task contained eight items (screenshots of intersections) that were the same for all participants, and the order was random. One point was awarded for each correctly identified turn. The maximum score was 8.

Pointing task. Participants were shown a viewpoint in the environment (a screenshot) and asked to indicate the direction of a landmark in the environment that was not visible to them. The task consisted of six trials. Three viewpoints in the environment were shown as encountered in the learning phase, whereas three viewpoints were not aligned (e.g., showing a building from the front when it had been seen from the side during the learning phase). The landmarks were located behind, in front, and to the left or right of the participants’ heading direction. In answering each question, participants used a circle on a sheet of paper: the center of the circle represented the place where the participant imagined they were standing. They drew an arrow indicating the direction they were facing, followed by a second arrow indicating the landmark. For each pointing task item, we calculated the absolute degrees of error between the answers given by the participants and the right answer, ranging from a minimum of 0 to a maximum of 180 degrees of error.

**Figure 2:**
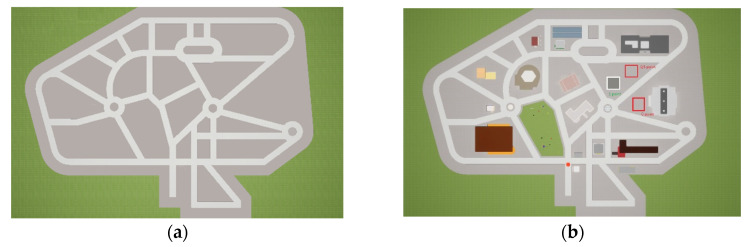
Sketched map used in map-completion task (**a**) and examples of scoring (0 point, 0.5 point, 1 point) for landmarks number 10 (lunch bar) in map-completion task (**b**).

Map-completion task. A sketched map of the virtual environment without landmarks, with a list of numbered landmarks, was shown to participants. The task consisted of placing each landmark on the sketched map by writing its corresponding number. Each landmark was in one of the gray areas of the map resulting from the intersections of roads in the road network (see Figure 2a). For each landmark, we assigned 0 points if the participant did not place the landmark in the correct gray area. Additionally, 0.5 points were awarded if the person located the landmark in the correct gray area but placed it incorrectly (not exactly in the correct position within the gray area). Finally, 1 point was awarded if the landmarks were placed in both the correct gray areas and positions within those areas (for a graphical example of scoring, see Figure 2b). The maximum score was 19. Two independent judges scored participants’ performance to obtain the final scores (sum of landmarks correctly located). Given the strong degree of accordance in their ratings (*r_s_* = 0.99), the first judge’s scores were used in the analyses.

#### 2.2.4. Self-Efficacy Manipulation

In the positive feedback condition, participants received feedback after three visuospatial tasks (route, survey, and map memory tasks). Specifically, a summary of the tasks appeared onscreen: for each task, we displayed a high fictitious (but plausible) score and an indication that the participant’s performance was above or significantly above average. Moreover, a summary comment on the participant’s performance appeared at the bottom of the page: “profile obtained: very good orientation abilities.” The feedback was presented for a few minutes as needed for each participant to carefully read the scores and comments.

In the neutral condition, participants also received feedback after the three cognitive tasks. In this case, however, the summary of the tests that appeared onscreen only showed that the tasks had been completed, without any further information on performance.

At the end of the experiment, as a manipulation check, participants in the positive feedback condition were asked (a) “Do you remember the feedback that has been given to you?” and (b) “Do you agree with the feedback?” to indicate how much they believed in the feedback (0 = *not at all* to 5 = *very much*).

### 2.3. Procedure

The experiment was run online (due to COVID-19 restrictions) using the Zoom platform. The experimenter met each participant in an individual session. The materials were presented using Qualtrics software and Google Jamboard. First, participants signed an informed consent explaining part of the study aims and their right to withdraw at any time. Then, participants completed a questionnaire of demographic questions and three spatial self-assessment questionnaires on sense of direction (SDSR), spatial anxiety (SAS), and spatial self-efficacy (global), respectively. The spatial self-assessment measures were presented in randomized order. Subsequently, participants performed the three visuospatial tasks (route task, survey task, and map memory task). Participants were randomly assigned to the neutral or positive feedback conditions. Subsequently, a video of a route within the virtual environment was shown to the participants twice. Then, they performed (a) the route-retracing task, (b) the pointing task, and (c) the map-completion task, in that order. Before each spatial-recall task, participants were asked to evaluate their degree of self-efficacy, such as how well they felt able to perform the task. Finally, manipulation check questions and debriefing were administered at the end of the study session.

## 3. Data Analysis

We conducted data analysis using RStudio. A total of 18 participants in the positive feedback condition were excluded from the analysis because they did not agree with the experimental feedback, having rated the question “Do you agree with the feedback that has been given to you?” with *not at all*, *barely*, or *slightly*. Thus, the final sample consisted of 213 participants: 99 participants in the positive feedback condition and 114 participants in the neutral feedback condition. Before removing the 18 participants, we checked whether they differed in terms of global self-efficacy from the other participants. The three groups (1 = positive feedback, 2 = control, and 3 = 18 participants who did not agree with the feedback) did not show significant difference between each other (*p* > 0.06) in terms of global spatial self-efficacy. 

First, to analyze the effect of self-efficacy manipulation on spatial-recall performance and task-specific self-efficacy, we ran independent sample *t*-tests comparing the means of the two groups. To further investigate the self-efficacy manipulation, we ran three mediation analyses using the Lavaan statistical package [26] to ensure simultaneously analysis of the relationships among the experimental manipulation (condition), task-specific spatial self-efficacy, and spatial-recall performance.

Second, to study the relationships between individual spatial factors (visuospatial tasks and spatial self-assessments) and spatial-recall task performance, we carried out generalized linear and binomial regression models on route retracing, pointing, and map completion. In each model, participants and the items of spatial-recall tasks were set as random effects. We adopted a stepwise approach based on Akaike Information Criterion (AIC; [27]). Specifically, predictors were added as follows: first, we considered a null model (m0) to be one that includes only intercepts without predictors; second, we explored the influence of the manipulation of self-efficacy (m1); and, third, we considered sex to be related to spatial performance (m2; [20]). Afterward, we added the three measures of visuospatial abilities (m3, m4, and m5; [5]). Next, we added SAS (m6) and SDSR scores (m7; [10]). Finally, after controlling for individual differences, we added global spatial self-efficacy (m8) and task-specific self-efficacy (m9) to determine their relationships with spatial performance. We entered the predictors into the model one at a time and kept each predictor only if it decreased the AIC of at least two units [28]. If adding the predictor did not decrease the AIC, its presence was considered negligible, and it was not considered in the subsequent model.

## 4. Results

Table 1 shows the means and standard deviations of the dependent variables route-retracing task, pointing task, and map-completion task, as well as the task-specific self-efficacy related to spatial-recall tasks (i.e., route-retracing, pointing, and map-completion tasks). Results from the *t*-tests showed no differences in spatial-recall tasks between the two groups, suggesting that the feedback did not produce differences in the spatial-recall tasks. However, differences arose between the two groups in all the task-specific self-efficacy questions before the spatial-recall tasks. This indicated that participants who received feedback reported higher levels of self-efficacy.

### 4.1. Self-Efficacy Manipulation and Task-Specific Self-Efficacy on Spatial-Recall Tasks

For each spatial-recall task, we ran a mediation analysis considering participant condition (1 = neutral feedback, 2 = positive feedback) as the independent variable, task-specific self-efficacy as a mediator, and performance in the spatial-recall task as the dependent variable.

**Figure 3:**
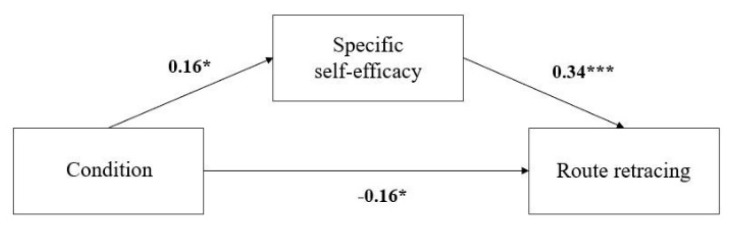
Mediation models of the effect of positive feedback on performance in route retracing. * *p* < 0.05; *** *p* < 0.001.

Route retracing. We found (a) a significant positive relation between participant condition and task-specific spatial self-efficacy (β = 0.16, *p* = 0.02), (b) a significant positive relation between task-specific self-efficacy and route retracing (β = 0.34, *p* < 0.001), and (c) a significant negative relation between the condition and route retracing (β = −0.16, *p* = 0.02). Finally, a significant positive indirect relationship between the condition and route retracing emerged through the mediation of task-specific self-efficacy (β = 0.05, *p* = 0.03, a × b; see Figure 3). In other words, receiving positive feedback negatively affected route-retracing performance and was mediated by task-specific self-efficacy.

**Figure 4:**
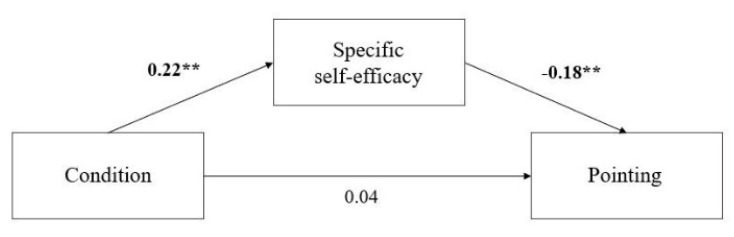
Mediation models of the effect of positive feedback on performance in pointing. ** *p* < 0.01.

Pointing. We found (a) a significant positive relation between the condition and task-specific spatial self-efficacy (β = 0.22, *p* = 0.001), (b) a significant negative relation between task-specific self-efficacy and pointing (β = −0.18, *p* = 0.008), and (c) a nonsignificant positive relationship between the condition and pointing (β = 0.04, *p* = 0.52). Finally, a significant negative indirect relationship between the condition and pointing mediated by task-specific self-efficacy emerged (β = −0.04, *p* = 0.04, a x b; see Figure 4). In other words, receiving positive feedback negatively influenced pointing performance and was mediated by task-specific self-efficacy.

**Figure 5:**
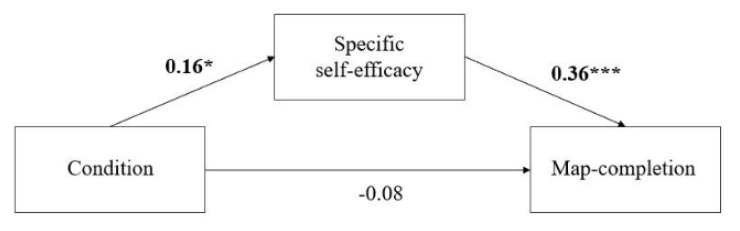
Mediation models of the effect of positive feedback on performance in map completion. * *p* < 0.05; *** *p* < 0.001.

Map completion. We found (a) a positive direct relationship between the condition and task-specific self-efficacy (β = 0.16, *p* = 0.02), (b) a significant positive relation between task-specific self-efficacy and map completion (β = 0.36, *p* < 0.001), and (c) a nonsignificant negative relationship between the condition and map completion (β = −0.09, *p* = 0.18). Finally, a significant indirect relationship between the condition and map completion emerged that was mediated by task-specific self-efficacy (β = 0.06, *p* = 0.03, a × b; see Figure 5), i.e., receiving positive feedback was negatively related to route-retracing performance and was mediated by task-specific self-efficacy.

### 4.2. Individual Visuospatial Factors and Spatial-Recall Tasks

Table 2, Table 3 and Table 4 show the results of model comparison relative to route-retracing, pointing, and map-completion tasks, respectively, following the model selection procedure explained in the Data Analysis section. We applied mixed linear and binomial models in which the condition (positive vs. neutral feedback), gender, visuospatial abilities tests, and self-reported measures were added, as all these variables are related to spatial-recall performance after learning in a virtual environment. Finally, after the condition and individual spatial factors, global and task-specific self-efficacy were added to investigate their influence after accounting for individual spatial factors and feedback.

For route retracing (see Table 2), the final model (m9) containing route task, map memory, and task-specific self-efficacy showed a statistically significant effect of visuospatial abilities, such as route task (odds ratio [OR] = 1.28, 95%, confidence intervals CI [1.10, 1.48], *p* = 0.001), map memory (OR = 1.30, CI [1.12, 1.50], *p* < 0.001), and task-specific self-efficacy (OR = 1.40, CI [1.20, 1.63], *p* < 0.001). Overall, the predictors explained 33% of the variance. The explained marginal variance was 6%.

For pointing (see Table 3), our final model (m7) containing route task, map memory, and sense of direction showed a statistically significant effect of visuospatial abilities, such as route task (β = −5.14, CI [−8.17, −2.11], *p* = 0.001), map memory (β = −3.71, CI [−6.76, −0.68], *p* = 0.017), and sense of direction (β = −4.78, CI [−7.82, −1.81], *p* = 0.002). Overall, the predictors explained 12% of the variance. The explained marginal variance totaled 3%.

For map completion (see Table 4), our final model (m9) containing route task, survey task, map memory, spatial anxiety, sense of direction, and task-specific self-efficacy showed statistically significant effects of route task (β = 0.04, CI [0.01, 0.07], *p* = 0.004), map memory (β = 0.03, CI [0.00, 0.06], *p* = 0.031), sense of direction (β = 0.03, CI [0.00, 0.07], *p* = 0.029), and task-specific self-efficacy (β = 0.06, CI [0.03, 0.09], *p* < 0.001). Overall, the predictors explained 32% of the variance. The explained marginal variance totaled 6%.

## 5. Discussion

We investigated whether experimental manipulation of self-efficacy through feedback after visuospatial tasks affects performance in virtual environment navigation-based learning and subsequent recall. We also investigated whether experimental manipulation through feedback could influence the performance of environmental tasks via self-efficacy. Finally, we examined the role of individual visuospatial factors in spatial-recall tasks; that is, regardless of the experimental condition, we wanted to determine the roles of visuospatial cognitive abilities and self-reported spatial abilities in spatial-recall task performance after virtual environment navigation learning.

To address these issues, we gave two groups of young adults feedback after three visuospatial tasks. One group received positive feedback indicating that their performance was above average, whereas the second group received neutral feedback indicating only that they had completed the tasks. Afterward, participants learned a route within a virtual environment and then recalled the environment while performing various tasks (route retracing, pointing, and map completion). To explore task-specific self-efficacy related to environmental learning, before each environmental task, we asked the participants to evaluate how well they felt able to accomplish the task in the virtual environment. Sense of direction, spatial anxiety, and global spatial self-efficacy were measured using questionnaires before the experimental manipulation. We hypothesized that the group that received positive feedback after visuospatial tasks would perform better in subsequent environmental recall tasks in the virtual environment. Furthermore, we hypothesized that task-specific self-efficacy would mediate the relationship between receiving feedback and performance in virtual environmental recall tests. Contrary to our expectations, we found no differences between the positive and neutral feedback groups’ performance in any of the environmental recall tasks. However, we observed differences in task-specific self-efficacy, and the mediation models showed that receiving feedback indirectly affected performance in all three spatial-recall tasks, mediated by task-specific self-efficacy. In other words, receiving positive feedback is related to higher levels of self-efficacy in task-specific spatial-recall tasks; in turn, task-specific self-efficacy is positively related to performance in spatial-recall tasks. Therefore, giving feedback after visuospatial cognitive tasks may have increased participants’ subsequent beliefs in their ability to accomplish spatial tasks. As a result, people may be more likely to put more effort into succeeding at similar or more complex tasks. This finding is consistent with Peifer et al. (2020), who showed that specific self-efficacy mediates positive feedback in cognitive tasks. Our study used recall tasks assessing virtual environment navigation learning rather than an arithmetic task. However, it is worth mentioning that the effect of receiving positive feedback is detectable only through the mediation models; that is, there is no direct evidence when comparing groups. It is possible that feedback does not have a generalized effect on everyone but only on those who accept it: only these individuals increase their sense of self-efficacy and consequently increase performance. Feedback had no effect on performance except when the individuals adapted their self-efficacy to it. Regarding a methodological explanation of these results, the remote mode adopted in this study (due to the COVID-19 pandemic) may have negatively affected the credibility of the feedback. This can be better analyzed in future studies. Moreover, we found a statistically significant negative relationship between receiving positive feedback and performance in route retracing. This finding might seem contradictory to the mediation effect, which showed a positive effect of the feedback condition on route retracing mediated by specific self-efficacy. This might have happened because route retracing was the first task participants completed in the environmental learning phase and was done remotely; therefore, we reiterate that the relationship deserves further examination. Another future development could include giving truthful rather than fictitious feedback to participants or feedback that highlights effort in performing the task rather than a performance evaluation [16].

Concerning the role of visuospatial factors, the regression models showed that after accounting for condition and gender, the route and map memory tasks were significant predictors of performance in route retracing, pointing, and map completion. These results are in line with the literature, which shows the contribution of visuospatial abilities (e.g., mental rotation abilities and VSWM) [6]. We add the relationship between spatial visualization and environmental learning. Visuospatial abilities in fact seem to sustain environmental spatial recall if the task maintains the same perspective (route retracing) or requires the ability to create a survey-like mental representation (pointing and map completion). As for self-reported spatial abilities, although global spatial self-efficacy measured via questionnaire was not significant, we found that task-specific self-efficacy measured before each task was a statistically significant predictor of one’s ability to retrace a path and represent a spatial mental representation of the environment. These results indicate that among the self-reported spatial abilities, motivational factors such as self-efficacy could be involved in spatial-recall performance. Furthermore, not global spatial self-efficacy in everyday spatial situations but rather the belief in one’s ability to accomplish a task, taking into consideration the specific context in which the task takes place (i.e., task-specific spatial self-efficacy), can influence the ability to learn and recall a virtual environment. In addition to self-efficacy, sense of direction is another subjective predictor in the map-completion task, confirming its role in the ability to create a spatial mental representation of the environment.

Some limitations of the present study should be acknowledged. We decided not to add a negative condition (in addition to the positive and neutral condition) for ethical reasons—that is, to avoid arousing negative emotions and affects among participants (e.g., anxiety). However, the positive feedback might differ from the neutral one in that it provides information with a positive valence, and this might make less clear whether the differences between the two conditions are related to the feedback itself or the valence of this information. To disentangle this issue, future studies could provide as neutral feedback the information that participants performed “average” in the neutral condition (more positive valence) in comparison to positive feedback.

In conclusion, our findings suggest that receiving feedback is related to performance and is mediated by self-efficacy. Receiving positive feedback is related to greater self-efficacy, which in turn is related to better performance in environmental learning through virtual environment navigation. Beyond experimental manipulation and gender, visuospatial abilities and sense of direction influence performance in spatial-recall tasks. Our results also underline the importance of considering not only global spatial self-efficacy, but also task-specific measures related to environmental learning. Therefore, motivational aspects and self-efficacy require further investigation in the spatial cognition domain.

## Figures and Tables

**Table 1:** Independent sample *t*-test.

	Positive Feedback Condition (*n* = 99)		Neutral Feedback Condition (*n* = 114)				
	*M*	*SD*	*M*	*SD*	*t*	*p*	Cohen’s *d*
Spatial-recall tasks							
Route retracing (accuracy)	6.46	1.20	6.70	1.26	1.49	0.14	0.21
Pointing (errors)	62.74	22.11	62.52	24.58	−0.07	0.95	−0.01
Map completion (accuracy)	5.31	4.37	5.56	4.42	0.41	0.68	0.06
Task-specific self-efficacy (Route retracing)	63.08	21.50	55.95	21.51	−2.42	0.02	−0.33
Task-specific self-efficacy (Pointing)	56.83	22.47	46.83	22.11	−3.27	0.001	−0.45
Task-specific self-efficacy (Map completion)	55.72	23.10	47.97	22.90	−2.45	0.02	−0.34

**Table 2:** Route retracing: stepwise model selection results. Variables that decreased AIC appear in bold.

Model	Route Retracing	AIC
m0	Participant + item	1428
m1	+ Condition	1428
m2	+ Sex	1430
**m3**	**+ Route task**	**1418**
m4	+ Survey task	1420
**m5**	**+ Map memory**	**1401**
m6	+ Spatial anxiety (SAS)	1559
m7	+ Sense of direction (SDSR)	1559
m8	+ Global spatial self-efficacy	1403
**m9**	**+ Task-specific self-efficacy**	**1385**

**Table 3:** Pointing: stepwise model selection results. Variables that decreased AIC appear in bold.

Model	Pointing	AIC
m0	Participant + item	13426
m1	+ Condition	13428
m2	+ Sex	13426
**m3**	**+ Route task**	**13416**
m4	+ Survey task	13416
**m5**	**+ Map memory**	**13411**
m6	+ Spatial anxiety (SAS)	13412
**m7**	**+ Sense of direction (SDSR)**	**13403**
m8	+ Global spatial self-efficacy	13405
m9	+ Task-specific self-efficacy	13403

**Table 4:** Map completion: stepwise model selection results. Variables that decreased AIC appear in bold.

Model	Map Completion	AIC
m0	Participant + item	3943
m1	+ Condition	3945
m2	+ Sex	3942
**m3**	**+ Route task**	**3939**
**m4**	**+ Survey task**	**3936**
**m5**	**+ Map memory**	**3928**
**m6**	**+ Spatial anxiety (SAS)**	**3925**
**m7**	**+ Sense of direction (SDSR)**	**3914**
m8	+ Global spatial self-efficacy	3914
**m9**	**+ Task-specific self-efficacy**	**3902**

## Data Availability

The data presented in this study are available on request from the corresponding author.
